# How Does Food Addiction Relate to Obesity? Patterns of Psychological Distress, Eating Behaviors and Physical Activity in a Sample of Lebanese Adults: The MATEO Study

**DOI:** 10.3390/ijerph182010979

**Published:** 2021-10-19

**Authors:** Anna Brytek-Matera, Sahar Obeid, Marwan Akel, Souheil Hallit

**Affiliations:** 1 Institute of Psychology, University of Wroclaw, 50-527 Wroclaw, Poland; 2 Faculty of Arts and Sciences, Holy Spirit University of Kaslik (USEK), Jounieh, Lebanon; saharobeid23@hotmail.com; 3 School of Pharmacy, Lebanese International University, Beirut, Lebanon; marwan.akel@liu.edu.lb; 4 Faculty of Medicine and Medical Sciences, Holy Spirit University of Kaslik (USEK), Jounieh, Lebanon; souheilhallit@hotmail.com; 5 Research Department, Psychiatric Hospital of the Cross, Jal Eddib, Lebanon

**Keywords:** food addiction, obesity, psychological distress, eating behaviors, physical activity

## Abstract

Food addiction is currently not an official diagnosis (as a standalone disorder substance-related and addictive disorder) in the Diagnostic and Statistical Manual of Mental Disorders, 5th Edition (DSM-5). To best of our knowledge, there is no previous research on differences between addictive-like eating behavior regarding its associations with psychological distress, eating behaviors and physical activity among individuals with obesity. The objective of the present study was to distinguish psychological and behavioral patterns of individuals with obesity concerning food addiction using a cluster analysis. We determined the profiles of the participants in terms of psychological distress, eating behaviors and physical activity and evaluated their association with food addiction. A cross-sectional study was conducted between September and November 2020, during the lockdown period imposed by the government for the COVID-19 pandemic. A sample of 507 individuals with obesity aged between 18 and 65 years participated in the present study by filling in the online questionnaire, including the validated Arabic version of the modified version of the Yale Food Addiction Scale, the Arabic version of the Depression, Anxiety and Stress Scale, the Three-Factor Eating Questionnaire, and the short version of the International Physical Activity Questionnaire. A cluster analysis was performed using the K-mean method to identify and group participants according to their patterns/profiles. A stepwise linear regression was conducted, taking the food addiction score as the dependent variable. Higher levels of uncontrolled eating, emotional eating and stress were significantly associated with higher food addiction score. Belonging to cluster 2 (psychological well-being and cognitive restraint) (B = 14.49) or cluster 3 (moderate psychological distress and cognitive restraint) (B = 6.67) compared to cluster 1 (psychological well-being, appropriate physical activity levels and eating behaviors) was significantly associated with higher food addiction score. The present research revealed that food addiction is significantly associated with higher psychological distress and maladaptive eating behaviors. Higher levels of uncontrolled eating, emotional eating and stress as well as belonging to clusters 2 and 3 were found to be predictors of food addiction in individuals with obesity in the present study. This knowledge could be useful in regard to psychological treatment of obesity and addictive-like eating behavior.

## 1. Introduction

Inappropriate dietary patterns, low levels of physical activity combined with high levels of sedentary behaviors and an increasingly obesogenic environment promoting poor dietary behaviors [1] are regarded as principal factors contributing to obesity [2]. Certain highly processed foods may have an addictive potential and that obesity may stem from an addictive response to these foods [3,4]. Highly processed foods, with added fats and/or refined carbohydrates are related to greater reported loss-of-control consumption and liking, pleasure, and craving. Thus, these foods may be particularly reinforcing and capable of triggering an addictive-like response in some individuals [5].

Food addiction is defined as hedonic eating behavior including the consumption of highly palatable foods in quantities beyond homeostatic energy requirements [6]. Food addiction is characterized by increased consumption of food as a result of food cravings to reach a state of pleasure [7]. Hebebrand et al. recognized the fact that it is difficult to differentiate between persons who overeat because of increased hunger and/or a reduced satiety from those with an “eating addiction”, with more in-depth studies needed to unravel these differences [8]. There are three clinical features evidenced in food addiction: (1) feelings of deprivation when food is withheld, (2) a propensity to relapse during periods of abstinence and (3) consumption that persists despite awareness of negative health, social, financial and other consequences [9,10]. According to more than forty studies [11], the prevalence of food addiction ranges from 5% in the general population [12,13] to over 40% among individuals with obesity [14,15]. A recent meta-analysis [9] indicated that food addiction prevalence was double in women than that in men (12.2% and 6.4%, respectively) and was higher in adults older than 35 years compared to those younger than 35 years (22.2% and 17.0%, respectively).

Obesity is comorbid with food addiction. Previous findings [9] have shown that food addiction has been linked to the increase in Body Mass Index (BMI >25 kg/m^2^). Several studies have also demonstrated the relationship between food addiction and disordered eating [16], compulsive eating behavior [17], emotional eating [18,19], mood and anxiety disorders [20,21] and depression [22,23,24]. In addition, former results [9] have found that seven out of ten articles showed that individuals with addictive-like eating behavior exhibited symptoms of depression. Furthermore, two studies found a positive relationship between depression and the presence of food addiction. The evidence suggests that individuals with depression are more likely to trigger food addiction as an emotional regulation strategy [25]. Whereas, individuals with addictive-like eating behavior may turn to excessive food consumption as a coping strategy for increased emotional distress [26]. Existing studies showed that the presence of food addiction is not influenced by level of physical activity; however, participants reporting a high level of physical activity displayed more symptoms of food addictive behavior than those with low and moderate physical activity [27]. Contrary to these findings, another study [2] found that individuals with food addiction spend less time engaged in physical activity and more time sitting on weekends when compared to non-food-addicted individuals, which may lead to increased food cravings, which may then contribute towards the addictive process. Research into the interrelationships between addictive-like eating and physical activity among the sample with obesity is warranted taking into consideration the fact that physical activity may play an important role in regulating addictive-like eating behavior [27].

The scientific validity of food addiction as a mental disorder and addictive behavior is still under investigation [11]. Previous results showed a correlation between less physical activity and more food addiction among obese and non-obese persons [2]. To the best of our knowledge, there is no previous research on differences between addictive-like eating behavior regarding its associations with psychological distress, eating behaviors and physical activity among individuals with obesity. The objective of the present study was to distinguish psychological and behavioral patterns of individuals with obesity concerning food addiction using a cluster analysis. We determined the profiles of the participants in terms of psychological distress, eating behaviors and physical activity and also evaluated their association with food addiction. We hypothesized that food addiction will be related to higher psychological distress and maladaptive eating behaviors in individuals with obesity. We also hypothesized that less physical activity would be associated with more food addiction in obese people.

## 2. Materials and Methods

### 2.1. Participants and Procedure

The current study was part of a large cross-cultural project (conducted in Lebanon and Poland) focusing on the Multidimensional Approach to Eating and Obesity (the MATEO study). The present study was carried out between September and November 2020, during the SARS-CoV-2 pandemic, when lockdown procedures were implemented at different instances, and the measures taken by the Government of Lebanon used to change on a regular basis according to the severity of the COVID-19 situation. A sample of 507 individuals with obesity aged between 18 and 65 years participated in the present study by filling in the online questionnaire. The sample was recruited through a snowball technique, from different demographic backgrounds from the five governorates of Lebanon (Beirut, Bekaa, Mount Lebanon, South Lebanon and North Lebanon). Respondents were briefed about the objective of the study and assured of the anonymity of the response. Participation in this study was voluntary. The participant could withdraw from participating in the study at any time.

### 2.2. Minimal Sample Size Calculation

According to the G-power software, and based on an effect size f2 = 2%, an alpha error of 5%, a power of 80%, and taking into consideration 20 factors to be entered in the multivariable analysis, the results showed that a minimal number of 395 was needed.

### 2.3. Measures

Sociodemographic characteristics information. Data were collected using a background information sheet. The background information sheet asked for information on age, gender, educational level (primary, complementary, secondary, university), marital status (single, married, divorced, widowed). Information about self-reported anthropometric measurement (height and weight) of the participants was also collected, which allowed us to calculate the body mass index.

Assessment of food addiction. The validated Arabic version of the modified version of the Yale Food Addiction Scale (mYFAS) [12] was used in the present study. The mYFAS is composed of 9 core questions including 1 item from each of the symptom groups that compose the 7 diagnostic criteria (sample item “I feel sluggish or fatigued from overeating”) plus 2 individual items that assess the presence of clinical impairment and distress (sample item “My behavior with respect to food and eating causes significant distress”). If a person endorses at least 3 of the 7 dependence symptoms and meets the criterion for clinical significance, she/he meets food addiction status (same as the YFAS) [11,12]. In the present study, the Cronbach’s alpha of the mYFAS was 0.859.

Assessment of psychological distress. The Arabic version of the Depression, Anxiety and Stress Scale (DASS-21) [28] was used in the present study. The DASS-21 is a 21-item screening tool for identifying, differentiating and assessing depression (sample item “I felt that life was meaningless”), anxiety (sample item “I felt close to panic”), and stress (sample item “I felt nervous”). A “general distress” total score (the DASS-total score) range between 0 and 120, and each of the subscales may range between 0 and 42. A cutoff score of 60 is used for the total DASS-21 score and a cutoff score of 21 is used for the depression subscale [29]. These cutoff scores are labeled as “high” or “severe”. In the present study, the Cronbach’s alpha for the total DASS-21 was excellent (α  =  0.912).

Assessment of eating behaviors. The Three-Factor Eating Questionnaire (TFEQ-R18) [30] was used in the present study. The TFEQ-R18 (18 items) assesses cognitive restraint (the conscious adherence to inadequate and/or restrictive diets; sample item “I consciously hold back at meals in order not to gain weight”), uncontrolled eating (compulsive eating behaviors defined by certain behaviors and criteria that may demonstrate a lack of control around food such as eating uncontrollably even when not physically hungry or consuming food much more rapidly than normal; sample “I am always hungry enough to eat at any time”) and emotional eating (eating because of specific negative emotion; sample item “When I feel lonely, I console myself by eating”) [30]. Due to a lack of the Arabic version of the TFEQ-R18, a certified translator performed the forward translation (from English to Arabic) of this tool. A committee of experts including healthcare professionals and language professionals verified this translation. The backward translation (From Arabic to English) was performed by a native English-speaking translator, who was unaware of the notions of the scales and who is fluent in Arabic. Then, the committee matched the back-translated English questionnaire with the original English questionnaire to detect inconsistencies and solve discrepancies between the two versions. The forward–backward translation process was repeated to reduce all uncertainties. In the present study, the Cronbach’s alpha of the TFEQ-R18 was 0.805.

Assessment of physical activity. The short version of the International Physical Activity Questionnaire (IPAQ-SF) [31] was used in the present study. The IPAQ-SF (7 items) is used to estimate total weekly physical activity by weighting the reported minutes per week within three activity categories (walking, moderate-intensity and vigorous-intensity physical activity) by a MET energy expenditure estimate assigned to each category of activity: walking = 3.3 METs, moderate intensity physical activity = 4.0 METs and vigorous intensity physical activity = 8.0 METs. The weighted MET-minutes per week (MET·min·wk^−1^) were calculated as MET intensity × duration × frequency per week [31]: walking (MET·min·wk^−1^) = 3.3 × walking minutes × walking days; moderate (MET·min·wk^−1^) = 4.0 × moderate-intensity activity minutes × moderate days; vigorous (MET·min·wk^−1^) = 8.0 × vigorous-intensity activity minutes × vigorous-intensity days (IPAQ Group, 2005). The weighted MET-minutes per week were summed across three activity categories to produce a weighted estimate of total physical activity from all reported activities per week (MET·min·wk^−1^) [31]. The Arabic version of the IPAQ-SF is available on the IPAQ website [32,33].

### 2.4. Statistical Analysis

Statistical package for the Social Sciences (SPSS) v.25 was used for the data analysis. The normality of distribution of the mYFAS score was confirmed via a calculation of the skewness and kurtosis; values for asymmetry and kurtosis between -1 and +1 are considered acceptable in order to prove normal univariate distribution [34]. These conditions consolidate the assumptions of normality in samples larger than 300 [35]. A cluster analysis was performed using the K-mean method to identify and group participants according to their patterns/profiles. The Student *t*-test and ANOVA test were used when comparing two and three or more means. Effect sizes were calculated for each association; in terms of effect size/correlation, coefficient values of │0.1–0.23│, │0.24–0.36│, and >│0.37│ indicated small, moderate, and large effect sizes/correlations, respectively [36]. A stepwise linear regression was conducted, taking the food addiction score as the dependent variable; all variables that showed an effect size or correlation >│0.24│in the bivariate analysis were included in the final model as independent variables; this will allow for achieving parsimonious models [36]. Cronbach’s alpha was recorded for the reliability analysis of all scales. Significance was set at *p* < 0.05.

## 3. Results

### 3.1. Sociodemographic Characteristics of the Participants

The total sample was composed of 507 individuals with class I obesity (*M*_BMI_ = 30.35 ± 5.36 kg/m^2^). The mean age of the participants was 29.86 ± 13.32 years. The sociodemographic characteristics of the participants as well as their scores in food addiction (assessed using mYFAS), psychological distress (using DASS-21), eating behaviors (using TFEQ-R18) and physical activity (using IPAQ-SF) are summarized in Table 1. The percentage of participants with food addiction was 25.2% (*n* = 128).

### 3.2. Cluster Analysis

**Figure 1:**
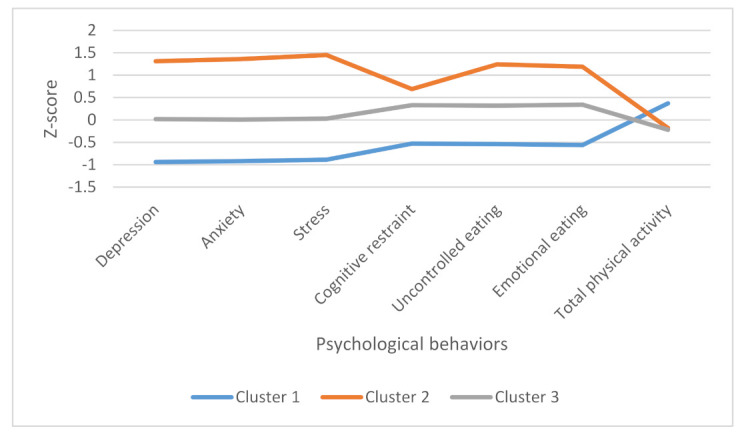
Profiles of individuals with obesity defined in the cluster analysis.

The cluster analysis categorized participants in three different profiles: cluster 1 named as “psychological well-being, appropriate physical activity levels and eating behaviors”, cluster 2 named as “psychological well-being and cognitive restraint”, and cluster 3 named as “moderate psychological distress and cognitive restraint” (Table 2, Figure 1).

### 3.3. Bivariate Analysis

Food addiction was found to be higher in participants belonging to cluster 2 (*n* = 77; 56.6%) compared to those belonging to cluster 1 (*n* = 6; 3.6%) and 3 (*n* = 45; 22.2%) (Table 3).

Moreover, higher psychological distress (depression, anxiety and stress) and maladaptive eating behaviors (cognitive restraint, uncontrolled eating and emotional eating) were significantly associated with a higher food addiction score. Whereas, higher levels of physical activity were significantly associated with a lower food addiction score (Table 4).

### 3.4. Multivariable Analysis

The results of the linear regression (Table 5, Model 1), taking the food addiction score as the dependent variable and each scale as an independent variable, showed that higher levels of uncontrolled eating (B = 0.56), emotional eating (B = 0.56) and stress (B = 0.25) were significantly associated with higher food addiction score.

The results of the linear regression (Table 5, Model 2), taking the food addiction score as the dependent variable and the clusters as an independent variable, showed that belonging to cluster 2 (B = 14.49) or cluster 3 (B = 6.67) compared to cluster 1 was significantly associated with a higher food addiction score

## 4. Discussion

The present study explored the typology of Lebanese adults with obesity based on psychological and behavioral patterns in regard to food addiction. In our study, three clusters emerged, consisting of “psychological well-being, appropriate physical activity levels and eating behaviors” (cluster 1), “psychological well-being and cognitive restraint” (cluster 2) and “moderate psychological distress and cognitive restraint” (cluster 3). Our findings showed that food addiction score was higher in individuals belonging to the cluster with lower levels of depression, anxiety and stress and higher levels of restrictive dietary intake to control weight (cluster 2) compared with the other two clusters. It is worth pointing out that, in a recent review [37] aimed to identify the symptoms of food addiction (“the signal”) from the more classic eating pathology (“restraint”) that can potentially elevate food addiction scores (“the noise”), the authors have recognized dietary restraint as a primary contributor of “noise” in the food addiction signal. Moreover, in the present study, higher psychological distress (depression, anxiety and stress) and maladaptive eating behaviors (cognitive restraint, uncontrolled eating and emotional eating) were associated with higher food addiction score. These results are in line with other studies showing that higher levels of depression are related to diagnosis of food addiction [12,38,39], as well as symptoms of food addiction [16,39,40]. The previous meta-analysis [38] also showed positive correlations between food addiction and anxiety and depression. In patients with obesity seeking bariatric surgery, food addiction was associated with a higher prevalence of mood and anxiety disorders, and loss of control over the consumption of high-fat, sugar and salt products [20].

Given the association of clusters 2 and 3 with food addiction, the presence of cognitive restraint might make a difference, more than the psychological well-being/moderate psychological distress. Restraint measures an individual’s cognitive effort to control their food intake to manage their body weight [39], and to engage in behaviors which favor a tighter regulation over energy intake, and subsequently body weight, with a lower tendency to overeat. This cognitive effort is likely to be particularly challenging when other external factors are present (for example, relationships, work–life balance, emotional fatigue) which may reduce the individual’s cognitive capacity to review and control their food intake, potentially leading to the goal of eating enjoyment being more easily prioritized [40]. Consequently, this then results in unhealthy eating patterns and weight gain [41,42,43,44]. 

Our results are consistent with the previous studies, which suggested that food addiction (both diagnosis and symptom score) was positively associated with both emotional and external eating [3,16,19]. A previous systematic review [25] has shown that individuals with food addiction were found to have a significantly higher intake of macronutrients (including fat and protein), starchy foods and takeaway food. Some food, like drugs of abuse, may be addictive and promote maladaptive eating patterns [26]. Highly processed foods (including sugar, fat, salt, caffeine, and flavor additives) with little nutritional value may be more likely to be capable of triggering an addictive process than healthy foods (e.g., fruits, vegetables, whole grains) [15]. Individuals with food addiction diagnosis were reported to have a significantly greater proportion of energy intake from fat and protein compared to individuals with no food addiction diagnosis [45]. The previous study showed that the relationship between food addiction and cognitive restraint was positive (but weak) or no relationship was found [15,46].

Our findings showed that a higher level of physical activity was significantly associated with lower food addiction score in individuals with obesity. Previous findings showed that participants reporting a high level of physical activity displayed more symptoms of food addictive behavior than those with low and moderate physical activity [27]. Contrary to these findings, another study [2] found that individuals with food addiction spend less time engaged in physical activity and present more sedentary behavior when compared to non-food-addicted individuals. Insufficient physical activity and sedentary behaviors may be the factors to consider in examining their relationship to food cravings [47]. They may lead to increased food cravings, which may contribute to the addictive process [2]. Research into the interrelationships between addictive-like eating and physical activity among samples with obesity is warranted, taking into consideration the fact that physical activity may play an important role in regulating addictive-like eating behavior [27]. A previous systematic review [48] showed that sedentariness was highly associated with food cravings, snacking and high-calorie snacking. In turn, individuals with addictive eating patterns experience more food cravings [49]. The difficulty to resist highly palatable, calorie-rich foods represents a special case of addictive behavior with similarities to other addictions (e.g., drug addiction) [50], which was confirmed by research showing that the responses to images of high-calorie foods to those responses others have observed among substance (ab)users to drug-related cues were similar.

### 4.1. Clinical Implications

Healthcare providers may gain insight from the findings of our study and help foster appropriate and effective programs to prevent excess weight problems among adults (such as personalized Cognitive Behavioral Therapy for Obesity CBT-OB). For example, underlying problems (psychological distress, lack of physical activity, maladaptive eating behaviors) may be tackled by the individuals through assistance from healthcare providers. In addition, it can be thought that performing awareness raising and encouraging studies in terms of the importance of healthy eating and regular exercise habits in the fight against obesity will decrease the incidence of obesity. Furthermore, food addiction is both an individual and collective health problem, and should be addressed at the societal level with broad policy interventions.

### 4.2. Limitations

Some limitations of our study need to be mentioned. In the present study, we used the mYFAS—a substitute for the YFAS (the most widely used measure of food addiction) [11]. Thus, participants with obesity could not meet the criteria for food addiction using the mYFAS (the items in the mYFAS were derived from a subset of the YFAS). Therefore, the use of the YFAS would be suitable for assessing the criteria of food addiction in our sample. Nevertheless, Lemeshow et al. (2016) [11] argued that the mYFAS may be an appropriate substitute for the YFAS and support the continued use of the YFAS and mYFAS to investigate whether the construct of food addiction is a valid psychiatric disorder. Participants might have not estimated their symptoms appropriately, and anthropometric measurements were self-reported, predisposing us to an information bias. A residual confounding bias is present since not all factors related to food addiction (such as the presence of chronic diseases) were taken into consideration in this study. A selection bias is present because of the snowball technique followed during the data collection; therefore, the study results might not be generalizable to the whole population. Finally, our data were collected during a lockdown period in Lebanon, where lifestyle changes were imposed on persons because of the containment measures, including, but not limited to, sedentary behaviors and modifications of other behaviors (eating, sleeping, smoking, etc.) [51,52].

## 5. Conclusions

Our findings showed that the prevalence of food addiction according to the mYFAS criteria was 25.2% in Lebanese individuals with obesity. Our study has revealed that food addiction is significantly associated with higher psychological distress and maladaptive eating behaviors, while higher levels of physical activity were related to low levels of food addiction. Higher levels of uncontrolled eating, emotional eating and stress, as well as belonging to the cluster named “psychological well-being and cognitive restraint” and the cluster named “moderate psychological distress and cognitive restraint”, were found to be predictors of food addiction in individuals with obesity in the present study. This knowledge could be useful concerning the psychological treatment of obesity and addictive-like eating behavior.

To sum up, our study provides insight into psychological and behavioral patterns of individuals with obesity concerning food addiction using a cluster analysis. These findings would allow the inclusion of psychological treatment among the cornerstones of obesity treatment, thus achieving a multidisciplinary approach in accordance to the multifactorial association of the obesity.

## Figures and Tables

**Table 1:** Sociodemographic characteristics of the participants with obesity (*n* = 507).

Variable	*n* (%) or Mean ± SD
Gender	
Male	220 (43.4%)
Female	287 (56.6%)
Marital status	
Single	297 (58.6%)
Married	190 (37.5%)
Widowed	13 (2.6%)
Divorced	7 (1.4%)
Education level	
Complementary or less	41 (8.1%)
Secondary	90 (17.8%)
University	376 (74.2%)
Food addiction status (mYFAS) ^a^	
No Yes	379 (74.8%) 128 (25.2%)
Age (in years)	29.86 ± 13.32
Body Mass Index (kg/m^2^)	30.35 ± 5.36
Depression (DASS-21)	17.85 ± 9.30
Anxiety (DASS-21)	17.29 ± 9.13
Stress (DASS-21)	14.24 ± 10.10
Cognitive restraint (TFEQ-R18)	15.92 ± 3.24
Uncontrolled eating (TFEQ-R18)	20.41 ± 5.90
Emotional eating (TFEQ-R18)	6.54 ± 2.37
Walking (MET·min·wk^−1^) (IPAQ-SF)	1723.74 ± 2709.23
Moderate intensity physical activity (MET·min·wk^−1^) (IPAQ-SF)	2026.73 ± 3491.85
Vigorous intensity physical activity (MET·min·wk^−1^) (IPAQ-SF)	6922.35 ± 13,402.47
Total physical activity (MET·min·wk^−1^) (IPAQ-SF)	10,672.83 ± 16,567.41

Note: ^a^ Food addiction status was based on presenting at least 3 of the 7 dependence symptoms and meets the criterion for clinical significance [12]; mYFAS = the modified version of the Yale Food Addiction Scale; DASS-21 = the Depression, Anxiety and Stress Scale; TFEQ-R18 = the Three-Factor Eating Questionnaire; IPAQ-SF = the short version of the International Physical Activity Questionnaire; MET·min·wk^−1^ = MET-minutes per week.

**Table 2:** Cluster analysis results among Lebanese sample with obesity.

Variable	Cluster 1 (*n* = 161)	Cluster 2 (*n* = 144)	Cluster 3 (*n* = 202)
Depression (DASS-21)	−0.94	1.31	0.02
Anxiety (DASS-21)	−0.92	1.36	0.01
Stress (DASS-21)	−0.89	1.45	0.03
Cognitive restraint (TFEQ-R18)	−0.53	0.69	0.33
Uncontrolled eating (TFEQ-R18)	−0.54	1.24	0.32
Emotional eating (TFEQ-R18)	−0.56	1.19	0.34
Total physical activity (IPAQ-SF)	0.37	−0.18	−0.22

Note: mYFAS = the modified version of the Yale Food Addiction Scale; DASS-21 = the Depression, Anxiety and Stress Scale; TFEQ-R18 = The Three-Factor Eating Questionnaire; IPAQ-SF = the short version of the International Physical Activity Questionnaire. Numbers represent regression coefficients for each factor in each cluster.

**Table 3:** Bivariate analysis of categorical variables associated with food addiction.

Variable	Mean ± Standard Deviation	*p*	Effect Size
Gender		0.548	0.053
Male	19.66 ± 8.41		
Female	20.11 ± 8.50		
Marital status		0.204	0.118
Single/Widowed/Divorced	20.29 ± 8.82		
Married	19.30 ± 7.81		
Education level		0.125	0.265
Complementary or less	22.00 ± 8.61		
Secondary	20.70 ± 7.98		
University	19.50 ± 8.53		
Clusters		<0.001	2.14
Cluster 1	13.14 ± 3.98		
Cluster 2	27.63 ± 8.09		
Cluster 3	19.81 ± 6.44		

**Table 4:** Bivariate analysis of continuous variables associated with the food addiction score.

Variable	Pearson’s Correlation Coefficient
Age (in years)	0.074
Depression (DASS-21)	0.586 ***
Anxiety (DASS-21)	0.602 ***
Stress (DASS-21)	0.636 ***
Cognitive restraint (TFEQ-R18)	0.369 ***
Uncontrolled eating (TFEQ-R18)	0.698 ***
Emotional eating (TFEQ-R18)	0.624 ***
Total physical activity (IPAQ-SF)	−0.237 ***

Note: DASS-21 = the Depression, Anxiety and Stress Scale; TFEQ-R18 = the Three-Factor Eating Questionnaire; IPAQ-SF = the short version of the International Physical Activity Questionnaire; *** *p* < 0.001.

**Table 5:** Multivariable analysis results (model 1 and model 2) among the Lebanese sample with obesity.

Model 1: Linear regression taking the food addiction score as the dependent variable and each scale as an independent variable.				
Variable	B	β	*p*	95% CI
Uncontrolled eating (TFEQ-R18)	0.56	0.39	<0.001	0.44-0.68
Stress (DASS-21)	0.25	0.30	<0.001	0.19-0.31
Emotional eating (TFEQ-R18)	0.56	0.16	<0.001	0.26-0.87
Variables entered in the model: Depression, Anxiety, Stress, TFEQ cognitive restraint, TFEQ uncontrolled eating, TFEQ emotional eating, Total IPAQ, Education level.				
Model 2: Linear regression taking the food addiction score as the dependent variable and the clusters as an independent variable.				
Variable	B	β	*p*	95% CI
Cluster 2 vs. cluster 1 ^□^	14.49	0.77	<0.001	13.06-15.92
Cluster 3 vs. cluster 1 ^□^	6.67	0.39	<0.001	5.36-7.99
Variables entered in the model: Education level, cluster 2, cluster 3.				

Note: DASS-21 = the Depression, Anxiety and Stress Scale; TFEQ-R18 = the Three-Factor Eating Questionnaire; B = Unstandardized Beta; β = Standardized Beta; ^□^ reference group.

## Data Availability

The authors do not have the right to share any data information as per the ethics committee rules and regulations.
